# Supplementary material for: Unconventionally fast transport through sliding dynamics of rodlike particles in macromolecular networks
Source: Nat Commun. 2024 Jan 15;15:525. doi: 10.1038/s41467-024-44765-7 (PMC10789817; doi:10.1038/s41467-024-44765-7)
Supplement: Supplementary file 1 — Supplementary Information [file 41467_2024_44765_MOESM1_ESM.pdf]

Supplementary Information

Unconventionally Fast Transport through Sliding Dynamics of Rodlike Particles in Macromolecular Networks

Xuanyu Zhang[1, 2, †], Xiaobin Dai[1, 2, †], Md Ahsan Habib[3, †], Lijuan Gao[1, 2], Wenlong Chen[1, 2], Wenjie Wei[1, 2], Zhongqiu Tang[3], Xianyu Qi[4], Xiangjun Gong[4], Lingxiang Jiang[3, *], and Li-Tang Yan[1, 2, *]

[1]State Key Laboratory of Chemical Engineering, Department of Chemical Engineering, Tsinghua University, Beijing 100084, China

[2]Key Laboratory of Advanced Materials (MOE), Tsinghua University, Beijing 100084, China
South China Advanced Institute for Soft Matter Science and Technology,

[3]School of Emergent Soft Matter, South China University of Technology, Guangzhou 510640, China

[4]Faculty of Materials Science and Engineering, South China University of Technology, Guangzhou 510640, China.

Table of Contents

I. Details of experiments of rods in macromolecular networks 3

1. Preparation of samples of synthetic networks and rods 3

2. Determination of trajectories of Au-NRs in the synthetic network. 4

3. Determination of the mesh size of PEGDA hydrogel. 6

II. Details of simulation models 8

1. Determination of coarse-grained molecular dynamics..8

2. Simulations pertinent to the experiments. 10

III. A theoretical model of a rod in the macromolecular network.........................12

IV. Sliding and hopping dynamics of a rod in the macromolecular network.......14

V. Rotational and off-axis dynamics of a rod in the macromolecular network....19

VI. Supplementary tables...23

VII. Supplementary figures..25

VIII. Supplementary References 37

I. Details of experiments of rods in macromolecular networks

1. Preparation of samples of synthetic networks and rods

The experimental network is polyethylene glycol diacrylate (PEGDA) network, which possesses excellent biocompatibility[1]. PEGDA network was prepared under ultraviolet (UV) irradiation[2,3]. 4% wt/vol solution of PEGDA (20 kDa, JenKem Technology USA) was prepared in distilled water and 0.02 g/ml lithium phenyl-2,4,6-trimethylbenzoylphosphinate (LAP) photo initiator was added. The solution was vortexed vigorously (Model: XH-D, Brand: Zigui, Shanghai), and then degassed by centrifuging (Model: TG16-WS, Brand: Cence, China) at 320 rpm for 5 min. The solution was cast on a 40 nm × 20 nm × 2 nm silicone slide with holes and cured under the UV light (wavelength 365 nm, 30 W) for 10 min. After the curing process, the PEGDA network was immersed under the distilled water for 48 h to remove the unreacted PEGDA monomers and allow the network to swell sufficiently. To calculate the mesh size of the network, we applied the methods developed by Peppas et al. [4,5], which has been widely used in designing macromolecular network systems as drug carrier[6] and artificial cytoskeleton[7]. The averaged mesh size of the PEGDA network was determined to be around 21.0 nm, as detailed in Table S2. With regard to the rods, PEG-capped Au nanorods (Au-NR) with the same diameter but different lengths were purchased from NanoSeedz Limited, Hong Kong and used for diffusion experiments without any modification. For monodispersing of the Au-NR in water, Au-NR solutions were sonicated (Model: 2800, Brand: Branson, China) for 20 min prior to put on the microscopic slide. To obtain the diameter and length of Au-NRs, around 2.5 μL Au-

NR sample was first diluted by dissolving in around 0.2 mL ethanol, sonicated for at least 20 min, and then dropped 3-6 μL solution on the transmission electron microscopy (TEM) copper grid. The copper grids were then stored at room temperature for at least 2 h to evaporate any solvent on it. Au-NR sizes were measured from the TEM images (Fig. S2) by Fiji (ImageJ) software8, and the averaged values of rod lengths used in the entire experiments are listed in Table S3. The diameter of these rods, including the surface chemistry with a small thickness of 3.5nm, was estimated to be around 19.1 nm.

2. Determination of trajectories of Au-NRs in the synthetic network.

To determine the trajectories of Au-NR in PEGDA network, we filled the network with 30 μL Au-NR solution, which was kept for 10-15 min on an optical microscope stage (Olympus BX51) at room temperature before observing the Au-NR diffusion into the PEGDA network[9]. For the trajectories of Au-NR in water, the microscopic slides were prepared almost in the similar way as mentioned above, but were filled with distilled water. The prepared slide was then kept on the microscope slides for at least 30 min before observing the trajectories. For analyzing the trajectories of Au-NR in both water and network, the images were taken by using dark field techniques[10]. For the water medium 400 images were recorded at a frequency of 20 Hz for 20 s time. On the other hand, for the hydrogel medium, total images were taken at a frequency of 10 Hz[11]. The general period for the measurement is 600s. About 30 individual starting time with separated period 10s are adopted for each time-averaged MSD. However, to give the experimental displacement probability distribution function (DPDF) with enough statistical significance and to exactly determine the diffusion coefficients, an extended

observation time up to 10800s, ensuring that at least 10 hopping events occur for the corresponding samples, was adopted for the calculation of DPDFs and MSDs, at L/a_x = 2.51±0.27, 2.63±0.31, 2.97±0.35. The spots were then detected and trajectories were obtained by the Trackpy software[12]. Briefly, the images were first loaded on the Fiji (ImageJ) software[8] and adjusted the brightness and contrast for better observation of the particles. To detect the particles in the image stack, Crocker-Grier algorithm was used[13]. Particles trajectories were then obtained by Nearest Neighbor Search tracker, as shown in Fig. S3. To eliminate the experimental error due to the cumulative vibration and drift of the microscope stage used for imaging during a longtime measurement, we compute the overall drifting motion, which will be subtracted away, adopting the reference frame of the particles' average position; this method can significantly reduce the creeping movement of the microscope and has been widely used elsewhere[14-16]. The tracking data was then exported in a CSV file format, and time-averaged MSDs, ensemble-averaged MSDs and diffusion coefficients were calculated, as shown in Figs. S3, 1b and 1c, respectively.

To delineate the longitudinal and transverse motions the rods in the experiments, the eigendecomposition algorithm is applied, which is based on principal component analysis algorithm[17,18]. For this purpose, we first calculate the gyration tensor of the trajectory of a rod,

$$\mathbf{S} = (S_{mn}) = \frac{1}{2N_{count}^2} \sum_{i=1}^{N_{count}} \sum_{j=1}^{N_{count}} (r_m^{(i)} - r_m^{(j)})(r_n^{(i)} - r_n^{(j)}) \qquad \text{(S1)}$$

where $r_m^{(i)}$ is the mth Cartesian coordinate of the position vector of the ith particle, and N_{count} is the total number of snapshots of a trajectory. The eigendecomposition of **S** gives the major and minor components of the gyration tensor,

$$\mathbf{S} = \mathbf{V}\boldsymbol{\Lambda}\mathbf{V}^T \tag{S2}$$

where $\boldsymbol{\Lambda} = diag(\lambda_{\|}, \lambda_{\perp})$ is the eigenvalue matrix, $\lambda_{\|}$, $\lambda_{\perp}$ are respectively the characteristic parameters of the trajectory in longitudinal and transverse directions, and $\lambda_{\|} \geq \lambda_{\perp} > 0$. $\mathbf{V} = [\mathbf{v}_{\|}, \mathbf{v}_{\perp}]^T$ denotes the eigenvector matrix, and $\mathbf{v}_{\|}, \mathbf{v}_{\perp}$ are the unit vectors in longitudinal and transverse directions, respectively.

3. Determination of the mesh size of PEGDA hydrogel.

To calculate the mesh size a_x of the hydrogel, we applied the Canal-Peppas mesh size mode[4,5], which had been widely used in designing hydrogel systems as drug carrier[5] and artificial cytoskeleton[6]. According to this model, the mesh size can be calculated by using hydrogel swelling ratio Q and modulus G. In order to obtain the swelling ratio of PEGDA hydrogel, 0.8 ml PEGDA solution with photo initiator was cast in a 35 mm petri dish and UV cured to get around 15 mm thickness of hydrogel. Then the hydrogel was carefully pill-off from the petri dish, rinsed in water and transferred to a large petri dish. After that the hydrogel was cut into a 25 mm circular size with a stainless still dice, and immersed in the deionized water for 48 h to measure the weight of the gel at swelling state ($m_{swollen}$). It should be noted that, during the swelling experiment water was replaced with the fresh water in every 12 h. Then the same hydrogel sample was slowly dried in a dryer to get dry weigh of gel (m_{dry}). Here,

we obtained the volume of the swollen network $V_{swollen}$, the volume of the dry network V_{dry}, the actual (not swollen) density of the PEG ρ = 1.12 g/mL, and the density of the water ρ_s = 18 g/mL. Volumetric swelling ratio can be calculated based on below equation:

$$Q = \frac{V_{swollen}}{V_{dry}} = \frac{m_{dry} / \rho + (m_{swollen} - m_{dry}) / \rho}{m_{dry} / \rho} = 43.7 \quad \text{(S3)}$$

Rheology test for as-prepared PEGDA hydrogel was performed on a rotational rheometer (ARES G2, TA Instruments, USA) at 25°C. Five different samples were measured to determine storage modulus G' and loss modulus G''. The complex shear modulus $G^* = G' + iG''$, but as the viscous contribution was negligible, shear modulus for all of samples were calculated by using $G = (|G'|^2 + |G''|^2)^{1/2}$. In detail, a parallel plate geometry of 20 mm diameter (PP 20) was used. As-prepared hydrogel cylinders were fixed between the plates. G was measured at an amplitude set within the linear elastic regime at constant frequency set to 1 Hz, and strain set to 1%. Based on G, the swelling ratio Q and a characteristic constant C_n, we estimated a theoretical mesh size by using the following equation[4],

$$a_x = l\sqrt{\frac{RTC_n\rho}{xM_rGQ}} \quad \text{(S4)}$$

where x is the number of binding for each chain (2 in case of PEGDA used in the experiments), ρ is the actual (not swollen) density of the repeating unit of the PEG, and M_r is the molar weight of the monomer (44g/mol). C_n is a characteristic constant ($C_n \approx$ 4 for PEGDA strands), and l is the distance between C-C backbone, $l \approx 0.154\times2$ nm for PEG. RT (kPa·mol^{-1}) at absolute temperature and is obtained from the universal gas constant, R. The calculation results are shown in Table S2.

II. Details of simulation models

1. Coarse-grained molecular simulations

In this paper, the transport of a rodlike particle in the macromolecular network is modeled by DPD method[19], which has been successfully used to model the structural and dynamic properties of macromolecular networks[20-22]. In the simulations, a bead represents a cluster of molecules and a set of interacting beads are considered. The time evolution is governed by Newton's equations of motion, $m\,\mathrm{d}\mathbf{v}_i/\mathrm{d}t=\mathbf{f}_i$. The force contains three parts, each of which is pairwise additive: $\mathbf{f}_i=\sum_j\left(\mathbf{F}_{ij}^C+\mathbf{F}_{ij}^D+\mathbf{F}_{ij}^R\right)$, where the sum runs over all beads j within a certain cutoff radius r_c. The conservative force is a repulsion acting along the line of bead centers, which is given by $\mathbf{F}_{ij}^C=a_{ij}(1-r_{ij}/r_c)\hat{\mathbf{r}}_{ij}$, where $\mathbf{r}_{ij}=\mathbf{r}_i-\mathbf{r}_j$ and $\hat{\mathbf{r}}_{ij}=\mathbf{r}_{ij}/\left|\mathbf{r}_{ij}\right|$. a_{ij} is a maximum repulsion between bead i and bead j, which has a linear relationship with Flory−Huggins parameter χ: $\chi_{ij}\approx\left(a_{ij}-a_{ii}\right)/3.27$ [19]. The interaction between like species a_{ii} is set as 25[19]. To bring out the entropic nature due to the conformational penalty of polymer strands deformed by a thick rod, the rod-strand interaction a_{rp} is set to be the same as that between like beads, that is, a_{rp}=25 otherwise noted, leading to $\chi_{rp}\approx 0$. Thus, based on the Flory−Huggins theory[23], only entropic items remain and the change of the free energy is governed by the entropy, capturing the physical nature of this system. The dissipative force and random force are given by $\mathbf{F}_{ij}^D=-\gamma\omega_D(r_{ij})\left(\hat{\mathbf{r}}_{ij}\cdot\mathbf{v}_{ij}\right)\hat{\mathbf{r}}_{ij}$ and $\mathbf{F}_{ij}^R=\sigma\omega_R(r_{ij})\theta_{ij}\hat{\mathbf{r}}_{ij}$, where ω_D and ω_R are $\mathbf{r}$-dependent weight functions and $\mathbf{v}_{ij}=\mathbf{v}_i-\mathbf{v}_j$. γ is a simulation parameter related to the viscosity arising from the interactions between the beads and $\sigma^2=2k_BT\gamma$, where k_B is the Boltzmann constant and T is the temperature.

θ_{ij} is a zero-mean Gaussian random variable of unit variance. These forces also act along the line of centers and conserve linear and angular momentum. Thus, we use $\omega_D(r_{ij}) = \omega_R(r_{ij})^2 = (1 - r_{ij}/r_c)^2$ for $r_{ij} < 1$. Since all of these forces conserve momentum locally, hydrodynamic behavior emerges. Additionally, the polymer strands are modeled as a sequence beads connected by harmonic bonds with an interaction potential given by $U_{\text{bond}} = K_b[(r\text{-}b)/r_c]^2$, where $K_b = 64k_BT$ is large enough to prevent bond crossing and $b = 0.5r_c$[24]. The factor k_BT is taken as the characteristic energy scale. In our simulations, $k_BT = 1$. The equations of motion are integrated in time with a modified velocity-Verlet algorithm[25] with time step of integration Δt. The characteristic time scale is then defined as $\tau = (mr_c^2/k_BT)^{1/2} = 1$. The remaining simulation parameter are $\gamma = 4.5$ and $\Delta t = 0.02\tau$ with a total bead number density of $\rho = 3$. The total simulation time is set as $t = 8 \times 10^4\tau$, which is long enough that mean square displacement (MSD) converges to linear dependence on time in logarithmic relationship.

To demonstrate the dynamics of a rod-like particle in the network, we choose a cubic box with dimensions of $42.76r_c \times 42.76r_c \times 42.76r_c$. The configuration of a network is taken to be a hexa-functional network, with periodic boundary condition in all directions. Such a network in this study consists of 46137 bonds and 2197 cross-links. The number of beads in a strand is 6, corresponding to the strand length of about $3.35r_c$. The total number of beads is 234549, leading to the total density $\rho = 3r_c^{-3}$. In these beads, the number of network beads is 42180 and the number of the solvent beads is 192369. Thus, the density of the network beads is about $0.54r_c^{-3}$.

Although mesh size of the network model is monodispersed, after a quantitative evaluation of the effects of the mesh size distribution on the dynamical behaviors, we find that the nonmonotonic dependence of the diffusivity on L for thick rods still keeps for the macromolecular networks which have been built with random mesh size distribution, indicating that the dynamical behaviors in the network within a certain scope of polydispersity can still fall into the physical principle revealed based on the regular network. Indeed, the simulation results based on this network capture the experimental results very well, as shown in Figs. 3b and S9.

A general particle-building model is adopted to build a set of rod particles fabricated by numbers of beads[26], and the rods move as rigid bodies with the Quaternion scheme[27], which is widely used in the simulations of the motion of rigid body and has been fully demonstrated to faithfully capture the transport of the nanoparticles[28]. Specifically, the rods are modeled as clusters of monomer sized beads, which have a diameter of $0.6r_c$. The rod is fabricated by arranging the beads on a triangulation surface with the inter-particle distance of $0.4r_c$, ensuring that the rod is not penetrated by other beads. By combining the linear and rotational physics into a single physics state and integrating, the motion of a rigid body in three dimensions can be simulated. The rod is originally located at the center of box and the principal axis of the rod is z-axis, as indicated in Fig. 2a. The network mesh size a_x, being essentially the correlation length between all pairs of strands comprising the networks (Fig. 2a), is fixed at about $a_x = 3.35r_c$. Particularly, d is set to be comparable to the mesh size, ranging from $1.3a_x$ to $1.9a_x$, and L ranges from $1.5a_x$ to $4.4a_x$, with which the off-axis dynamics is negligible

(see Section V for more details). The normalized sizes d/a_x and L/a_x are used, representing the size matching between a rod and a network mesh.

2. Simulations pertinent to the experiments.

To evaluate effectiveness of the simulation model in the experimental systems, we perform calculation based on the specific experiments and compare our simulation outcome directly with the experimental results. Specifically, the experimental system is selected as a rod with d = 20nm and L = 40 nm in the PEGDA network. We relate the simulation parameters of physical length and time scales through the values of the collective diffusion coefficient of the rod[20]. The length r_c can be considered as the side of a cube containing an averaged of ρ beads[25]. Therefore, $r_c = (\rho V_b)^{1/3}$, where V_b is the volume of a bead. Here, we choose the sizes of the rod as d = 20 nm and L = 40 nm, and the rod consists of N_m = 251 beads in our simulations. Thus, the volume of the rod $V_r = \pi d^2 L/4 = 1.26 \times 10^4$ nm^3, and V_b is around 50.2 nm^3. Because the bead density ρ is 3, a cube of r_c^3 contains three beads and therefore corresponds to a volume of 158nm^3. Thus, the physical size of the interaction radius, r_c, is roughly estimated as 5.32 nm.

To measure the viscosity of our experimental systems, i.e., nanorod in the polymer solution, by performing additional particle tracking experiments of this nanorod in 4% PEGDA solution. The rod is considered as an equivalent sphere in the follow calculation, and thus the hydrodynamic diameter of the rod can be estimated as $r_h = (3Lr^2/4)^{1/3}$ = 22.9nm. As shown in Fig. S10, the MSD of the nanorod in the polymer solution exhibits the normal diffusion, with diffusion exponent 1 as expected at all time scale. Using a linear regression in log space, we can easily fit this ensemble MSD to a

power law, $\langle \Delta z^2(t) \rangle = At$, where A = 0.0295μm^2/s. For 2-dimensional diffusion in the particle tracking experiment, the diffusion coefficient of the nanorod can be calculated as $D_{\exp}$ = A/4 = 0.0737μm^2/s. Using the Stokes-Einstein (SE) equation, $D_{\exp}$ = $k_B T/(6\pi\mu r_h)$, we can calculate the real viscosity of the nanorod,

$$\mu = \frac{k_B T}{6\pi D_{\exp} r_h} = 1.32\,\mathrm{Pa \cdot s} \tag{S5}$$

As proposed by Ref. [19,20], the diffusion constant of solvent beads in the DPD simulations, D_{calc} = $0.1707 r_c^2/\tau$, and the physical time scale of the simulation pertinent to the experiments can be roughly estimated as, $\tau = N_m D_{calc}/D_{\exp}$= 0.017s.

In order to keep our simulations pertinent to the experiments, we perform the corresponding simulations both in the network and neat solvent to get a set of D and D_0, where D is the longtime diffusion coefficient in the network and D_0 denotes that in neat solvent. To ensure that the experiments and the simulations are comparable, d/a_x is set as 1.05 and a set of L/a_x are selected as 1.5, 2.0, 2.2, 2.5 and 3.0. As shown in Fig. 2c, we obtain the normalized diffusion coefficients, D/D_0, of the simulation results, which are in good agreement with the experimental values.

III. A theoretical model of a rod in the macromolecular network

We start from the Deam-Edwards Hamiltonian of a Gaussian network with excluded volume of a rod[30],

$$H\left(\mathbf{R}_{ij}, \mathbf{r}_{\text{rod}}, \mathbf{l}_{\text{rod}}\right) = \frac{3k_B T}{2Nb} \int_0^1 \mathrm{d}s \left(\frac{\partial \mathbf{R}_{ij}(s)}{\partial s} \right) + U_{mr}(\mathbf{R}_{ij}, \mathbf{r}_{\text{rod}}, \mathbf{l}_{\text{rod}}) \tag{S6}$$

where $\mathbf{R}_{ij}$ is the path vector of the strand with its start $\mathbf{R}_{ij}(0)=\mathbf{r}_i$ and end $\mathbf{R}_{ij}(1)=\mathbf{r}_j$, $\mathbf{r}_{\mathrm{rod}}$, $\mathbf{l}_{\mathrm{rod}}$ are the position and directional vectors of the rod, $s\in[0,1]$ is the contour variable, N is the number of bonds in a strand, and b is the Kuhn length. The hard-core interaction between the monomer and rod is given by,

$$U_{mr}(\mathbf{R}_{ij},\mathbf{r}_{rod},\mathbf{l}_{rod})=\begin{cases}\infty & \|(\mathbf{R}_{ij}-\mathbf{r}_{\mathrm{rod}})\cdot\mathbf{l}_{rod}\|<L/2, \|(\mathbf{R}_{ij}-\mathbf{r}_{\mathrm{rod}})-(\mathbf{r}_{ij}-\mathbf{r}_{\mathrm{rod}})\cdot\mathbf{l}_{\mathrm{rod}}\|<d/2 \\ 0 & else\end{cases} \quad \text{(S7)}$$

where d, L are the diameter and length of the particle, respectively. By substituting the equations below, we get the partition function of the entire system[31],

$$Z\left(\mathbf{r}_{\mathrm{rod}},\mathbf{l}_{\mathrm{rod}}\right)=\prod_{k}\int \mathrm{d}\mathbf{r}_k\prod_{(i,j)}\int \mathcal{D}\mathbf{R}_{ij}\delta(\mathbf{r}_i-\mathbf{R}_{ij}(0))\delta(\mathbf{r}_j-\mathbf{R}_{ij}(1))\exp\left[-\beta H(\mathbf{R}_{ij},\mathbf{r}_{\mathrm{rod}},\mathbf{l}_{\mathrm{rod}})\right] \quad \text{(S8)}$$

where $k=\{\mathbf{r}_i\}_{i=1}^{M}$ is the set of cross-links containing M cross-links between the efficiently bridged Gaussian chains, and (i,j) represents linker connections with ends $\mathbf{r}_i$ and $\mathbf{r}_j$. The Helmholtz free energy of the system is given by,

$$F(\mathbf{r}_{rod},\mathbf{l}_{rod})=-k_B T\ln Z(\mathbf{r}_{rod},\mathbf{l}_{rod}) \quad \text{(S9)}$$

For Gaussian chains, the correlation function of $\partial\mathbf{R}_{ij}(s)/\partial s$ gives

$$\int ds\frac{\partial\mathbf{R}_{ij}(s)}{\partial s}\cdot\frac{\partial\mathbf{R}_{ij}(s')}{\partial s'}=\int ds\delta(s-s')\left\|\frac{\partial\mathbf{R}_{ij}(s)}{\partial s}\right\|^2 \quad \text{(S10)}$$

We thereby define the integral of a strand,

$$L_{ij}(\mathbf{R}_{ij},\mathbf{r}_{rod},\mathbf{l}_{rod})=\int_0^1 ds\frac{\partial\mathbf{R}_{ij}(s)}{\partial s}\Phi(\|(\mathbf{R}_{ij}-\mathbf{r}_{rod})\cdot\mathbf{l}_{rod}\|-L/2)\Phi(\|(\mathbf{R}_{ij}-\mathbf{r}_{rod})-(\mathbf{R}_{ij}-\mathbf{r}_{rod})\cdot\mathbf{l}_{rod}\|-d/2) \quad \text{(S11)}$$

where $\Phi(x)$ is the Heaviside step function.

As shown in Fig. S11, to calculate the integral of a strand $L_{ij}(\mathbf{R}_{ij},\mathbf{r}_{rod},\mathbf{l}_{rod})$ cross-linked by points P and Q at positional vectors $\mathbf{r}_i$ and $\mathbf{r}_j$, we consider an osculating plane A (green plane) coming across the points P, Q and O_0 with the normal vector $\mathbf{n}$. The path of the chain $\mathbf{R}_{ij}$ from P to Q in the plane A desires to be one of the

conformations of chains $\mathbf{R}_{ij}(s)$. Randomly selecting the normal vector $\mathbf{n}$ gives all conformations of chains that slip around the surface of rod. Here, the dihedral angle θ is defined as,

$$\theta = \arccos \frac{\mathbf{n} \cdot \mathbf{l}_{rod}}{\| \mathbf{n} \| \| \mathbf{l}_{rod} \|} \tag{S12}$$

so that the path integral $\int \mathcal{D}\mathbf{R}_{ij} f(\mathbf{R}_{ij})$ can be converted to $\int \mathrm{d}\theta \int \mathrm{d}\mathbf{n} g(\mathbf{R}_{ij}, \mathbf{n})$, where f and g are functions.

In the calculations, we apply the Graham scan algorithm[32] on the convex shape of the "untouched" area to evaluate the integral length $L_{ij}(\mathbf{R}_{ij}, \mathbf{r}_{rod}, \mathbf{l}_{rod})$ of path $\mathbf{R}_{ij}$ for various $\mathbf{n}$. Here, we study the condition that the strand contacts the surface of the rod. Its solution can be written as

$$\begin{cases} \| (\mathbf{r} - \mathbf{r}_{rod}) \cdot \mathbf{l}_{rod} \| < L/2 \\ \| \mathbf{r} - \mathbf{r}_{rod} - (\mathbf{r} - \mathbf{r}_{rod}) \cdot \mathbf{l}_{rod} \| < d/2 \end{cases} \tag{S13}$$

where $\mathbf{r} = \lambda \mathbf{r}_p + (1-\lambda)\mathbf{r}_q$ represents the points on line segment PQ, and $0 \le \lambda \le 1$. If contacts, the integral L_{ij} is determined by the intersection of the convex shape in plane A, taking the form

$$\begin{cases} \| (\mathbf{r} - \mathbf{r}_{rod}) \cdot \mathbf{l}_{rod} \| < L/2 \\ \| \mathbf{r} - \mathbf{r}_{rod} - (\mathbf{r} - \mathbf{r}_{rod}) \cdot \mathbf{l}_{rod} \| < d/2 \\ \| (\mathbf{r} - \mathbf{r}_{rod}) \cdot \mathbf{n} \| = 0 \end{cases} \tag{S14}$$

IV. Sliding and hopping dynamics of a rod in the macromolecular network

In this section, we provide a more detailed discussion regarding the dynamical regimes of Brownian, sliding, and hopping dynamics for a rod in the macromolecular

network. If n jumps have occurred up to time t, the distribution of the total number of the network cells traversed can accordingly be obtained in Fourier-Laplace space from the Montroll-Weiss equation[33],

$$S(k,s)=\frac{1-\tilde{\psi}(s)}{s\left[1-\hat{\phi}(k)\tilde{\psi}(s)\right]} \tag{S15}$$

where $\tilde{\psi}(s)$ is the Laplace transform of the waiting time distribution $\psi(t)$ and $\hat{\phi}(k)$ is the Fourier transform of the hopping length distribution $\phi(\tilde{z})$.

The waiting distribution $\psi(t)$ can be established on the basis of the long time asymptotic behaviour of the rod. Since the mean square displacements (MSD) of Brownian, sliding, and hopping dynamics recover back to the normal diffusion in the long-time scale in Fig. 2b, it can be approximated that the waiting process is a Poisson process.[34] The waiting time distribution of Poisson process in continuous time domain take the exponential form, which is given by

$$\psi(t)=\frac{1}{\tau_{hop}}\exp(-\frac{t}{\tau_{hop}}) \tag{S16}$$

In terms of Kramers' rate theory,[35] the well-separation time scales can be obtained and the averaged time of the waiting process can be described as,

$$\tau_{hop}=\frac{2\pi\gamma}{\sqrt{|k_s|k_l}}\exp(U_b/k_BT) \tag{S17}$$

where k_s and k_l are the curvatures of the potential along $\mathbf{z}$ -axis at the saddle point $\tilde{z}=0$ and the starting minimum $\tilde{z}=0.5a_x$, respectively.

The jumping length distribution $\phi(\tilde{z})$ relies on the free energy barrier U_b in different regimes, as discussed below:

(1) For the hopping dynamics, the particle spends most of the time close to the minimum free energy point in a network cell and only occasionally escapes to another one. It can be estimated that the hopping length is the integral multiple of the mesh size a_x with regard to the periodic conditions, and thus $\phi(\tilde{z})$ has the periodic distribution[36,37],

$$\phi(\tilde{z}) = CP(n)\delta(|\tilde{z}| - na_x) \tag{S18}$$

where a_x is the characteristic hopping length, $P(y) = \exp(-4\beta U_b y^2 / a_x^2)$ gives the Boltzmann distribution, U_b represents the energy barrier, n is an integral number, and C is the normalization constant such that $\sum_{n=0}^{\infty} CP(na_x) = 1$. By subtracting the Laplace transformation of equation (S16) $\tilde{\psi}(s) = 1/(1+\tau_{hop}s)$ and performing the inverse Laplace transformation of equation (S15), we can get the characteristic function $K(k,t)$ in the time domain,

$$K(k,t) = \exp\left[-\frac{t}{\tau_{hop}}(1-\tilde{\phi}(k))\right] \tag{S19}$$

For short time scales, $t \ll \tau_{hop}$, the hopping event doesn't happen, so that equation (S18) can be assumed by, $\phi(\tilde{z}) = CP(\tilde{z})\delta(\tilde{z})$. Substituting the Laplace transformation of that, it can be obtained by the first-order approximation,

$$K(k,t) = 1 - \frac{t}{\tau_{hop}}\left[1 - \exp\left(-\frac{k^2}{4\beta U_b / a_x^2}\right)\right] \tag{S20}$$

As a result, in position space we obtain

$$G_s(\tilde{z},t) = \delta(\tilde{z})\left(1 - \frac{t}{\tau_{hop}}\right) + \frac{t}{\tau_{hop}}\frac{a_x}{2\sqrt{2\pi\beta U_b}}\exp(-\frac{\tilde{z}^2}{16\pi\beta U_b / a_x^2}) \tag{S21}$$

In contrast, for long time scales, $t \gg \tau_0$, we perform saddle-point approximations around $k = 2\pi / a_x, \pi / a_x, 2\pi / 3a_x, \ldots$, and yields

$$K(k,t) = \exp\left[-\frac{t}{\tau_{hop}} k^2 a_x^2 \right] \tag{S22}$$

where the aliased characteristic function K is defined on the interval $(-\pi / a_x, \pi / a_x]$ [37]. The displacement distribution $G_s(\tilde{z},t)$ is thus evaluated by the convolution in each interval

$$G_s(\tilde{z},t) = \sum_{n=0}^{\infty} NP(n)\delta(|\tilde{z}| - na_x) \frac{1}{\sqrt{4\pi a_x^2 t / \tau_{hop}}} \exp\left(-\frac{\tilde{z}^2 \tau_{hop}}{4ta_x^2} \right) \tag{S23}$$

(2) For the sliding dynamics, to describe the non-local nature of irregular peaks in $G_s(\tilde{z},t)$, we apply the master equation for the random walk, which is given by

$$G_s(\tilde{z},t) = G_s(\tilde{z},0) + \int_0^t \sum_{\tilde{z}'} \omega(\tilde{z} - \tilde{z}') G_s(\tilde{z}',t') dt \tag{S24}$$

where $\omega(\tilde{z})$ is the kernel of the diffusion equation. Equation (S24) can be transformed into the discrete form,

$$G_s(\tilde{z},t) = \sum_{n=0}^{\infty} P(n,t) P(\tilde{z},n) \tag{S25}$$

where $P(\tilde{z},n)$ and $P(n,t)$ represent the probability density function (PDF) of the displacement and the number of hopping events happened.

We consider that at time *t*, the random variable of displacement X_t gives

$$X_t = \sum_{k=0}^{\infty} J_k \tag{S26}$$

where J_k is the jump length with its distribution $\phi(\tilde{z})$. Each jump can be seemed as a difference of two independent variables $J_k = J_k^+ - J_k^-$, where J_k^+, J_k^- denote forward and backward displacements between two neighboring cells with the same

distance L. According to the Poisson point measure[38], the probability of the distance between any two consecutive cells on this line is exponential, given by

$$p(L) = \frac{1}{a_x}\exp\left(-\frac{L}{a_x}\right) \tag{S27}$$

For a given cell with a fixed L, the distribution is uniform. In one dimension, it is $p(z\,|\,L) = 1/L$, $|z| < L/2$, yielding,

$$\phi(\tilde{z}) = \int_{|z|<L/2} p(L)p(\tilde{z}\,|\,L)dL = \frac{1}{a_x}\exp\left(-\frac{2|\tilde{z}|}{a_x}\right) \tag{S28}$$

which is found to be the Laplace (also called "two sided exponential") distribution with mean 0 and variance $a_x/2$, written as $J_k \sim Lap(0, a_x/2)$.

Then two compound Poisson processes $N_t^+ = \{T_1 + T_2 + ... + T_k, J_k^+\}$, $N_t^- = \{T_1 + T_2 + ... + T_k, J_k^-\}$ for the independent waiting time spans T_k are obtained, where $T_k \sim Poiss(2t/\tau_{hop})$ gives the standard Poisson distribution. We may thereby represent the total displacements as a difference of two positive independent processes, $X_t = X_t^+ - X_t^-$, where $X_t^\pm = \sum_{k=0}^{N_t^\pm} J_k^\pm$.

It can be proved that the sum of independent exponential distribution gives Gamma distribution, i.e., $\sum_{k=0}^{n} J_k^\pm \sim Gamma(n+1, 2/a_x)$. By substituting the PDF Poisson distribution $P_{N^\pm}(n,t) = \frac{(t/\tau_{hop})^n}{n!}\exp(-\frac{t}{\tau_{hop}})$ and the Gamma distribution $P_{X^\pm}(\tilde{z},n) = \frac{(2\tilde{z}/a_x)^n}{n!}\exp(-\frac{2\tilde{z}}{a_x})$, the PDF of $X_t^\pm$ gives,

$$P_{X^\pm}(\tilde{z},t) = \sum_{n=0}^{\infty} P_{N^\pm}(n,t)P_{X^\pm}(\tilde{z},n) = 2I_0\left(2\sqrt{2\tilde{z}t/a_x\tau_{hop}}\right)e^{-2\tilde{z}/a_x}e^{-t/\tau_{hop}} \tag{S29}$$

where I_0 is the modified Bessel function of the first kind. The PDF of X_t takes the convolution of equation (S29),

$$G_s(\tilde{z},t) = \int_0^\infty dx P_{X^+}(x+|\tilde{z}|,t) P_{X^-}(x,t) = 4e^{\frac{-2|\tilde{z}|}{a_x}} e^{\frac{-2t}{\tau_{hop}}} \int_0^\infty dx e^{-4x/a_x} I_0\left(\sqrt{\frac{8t(|\tilde{z}|+x)}{a_x \tau_{hop}}}\right) I_0\left(\sqrt{\frac{8tx}{a_x \tau_{hop}}}\right) \quad \text{(S30)}$$

(3) For the Brownian dynamics with $U_b = 0$, the jump length takes the form of Gaussian distribution,

$$\phi(\tilde{z}) = \frac{1}{\sqrt{4\pi a_x}} \exp\left(-\frac{\tilde{z}^2}{4a_x^2}\right) \quad \text{(S31)}$$

which exhibits normal diffusion at all times. Substituting equations (S16) and (S31) into equation (S15), and taking the inverse Fourier-Laplace transformation, we get

$$G_s(\tilde{z},t) = \frac{1}{\sqrt{4\pi a_x^2 t / \tau_0}} \exp\left(-\frac{\tilde{z}^2 \tau_0}{4a_x^2 t}\right) \quad \text{(S32)}$$

where τ_0 is the characteristic waiting time in solvents.

V. Rotational and off-axis dynamics of a rod in the macromolecular network

The rotation and off-axis dynamics of a rod significantly depend on its length as well as diameter[39-41]. In this work, the longitudinal and transverse motions are delineated by the eigendecomposition algorithm, which has been widely used in other systems with anisotropic particles[17,18]. To apply this algorithm, the dynamics of rods should satisfy the condition that the rotational dynamics plays a trivial role in the transport of the rods[42,43]. Thus, we first provide a strict examination of the simulation and experimental results regarding the rotational dynamics of the long and thick rods used in the present work. Figure 1c presents the representative trajectories for rods with

different lengths corresponding to the hopping and sliding dynamics. It can be identified that all trajectories form straight lines across the sample rather than randomly diffusing in 2D, indicating that over the ranges of rod length and diameter explored in the present work, the rotational event takes place in an extremely low probability and thereby has little effect on the longitudinal motion of the rods in the macromolecular networks. That is, within the whole temporal scales (over 600s) of simulation and experiments, almost no motion towards the direction normal to the major axis can be identified, indicating that the rotational event indeed takes place in an extremely low probability and thereby has little effect on the longitudinal motion of the rods in the macromolecular networks.

Next, to consolidate the trivial role of the rotational dynamics, we provide a fundamental discussion on the rotational and off-axis dynamics of a rod, through theoretically calculating the free energy barrier for the rod rotation in the macromolecular network and thereby estimating the waiting time for a rotational event. Theoretically, due to the confinement of the network strands, the rod should surmount an energy barrier when rotating in the network. Based on the theoretical model of a rod in the macromolecular network, as stated in the above section, we calculate the rotational energy barrier contributed by the local network strands, i.e., $\Delta E_{rot} = \Delta F_{max} - \Delta F_{min}$, where ΔF_{max} and ΔF_{min} denote respectively the maximum and minimum free energy in all the rod directions characterized by the parameter $\mathbf{n}_{rod}$ in equations (S6)-(S9). The results indicate that the longer and thicker of a rod, the higher energy it experiences when rotating, and for a typical rod used in the current

work, with diameter d / a_x = 1.0 and length L / a_x = 2.0, the energy barrier can be calculated as 10.3 $k_B T$. Furthermore, the waiting time for the rotational event can be determined by the Kramers' rate theory[35],

$$t_{hop} = t_{r0} \exp(\Delta E_{rot} / k_B T) \tag{S33}$$

where t_{r0} is the waiting time for rotating at $\Delta E_{rot} = 0$ and can be estimated in polymer solution. The rotational diffusion coefficient of the rod of d / a_x = 1.0 and length L / a_x = 2.0 in polymer solution can be calculated as

$$D_r = \frac{k_B T}{8\pi\mu r_h^3} = 10.03\text{s}^{-1} \tag{S34}$$

where the solvent viscosity is $\mu = 1.32\text{Pa}\cdot\text{s}$ and the hydrodynamic radius $r_h = 22.9\text{nm}$ (see Section II of Supplementary Information for more details). Thus, the waiting time in the pure solvent can be obtained, as $t_{r0} = 1 / D_r = 0.0997\text{s}$. Then, with equation (S33), the waiting time at $\Delta E_{rot} = 10.3 k_B T$ is realized, as

$$t_{hop} = 2.96 \times 10^3 \text{s} \tag{S35}$$

which is much larger than the observation time in experiment (600s). Actually, almost all the rod lengths used in our experiments are equal or even larger than L = 40nm, indicating that the rotational dynamics plays a trivial role in the transport of nanorods in these systems. Note that the only one exception is the rod with L = 30nm (L / a_x = 1.5) and the calculation of t_r for this system is about 200 s. Although this value is a little smaller, the rotational event take place in a low probability if considering the observation time of 600s, which, we believe, has a little effect on the longitudinal motion of this rod in the macromolecular network and cannot modify the physics of the whole experimental systems.

Third, establishing the condition of restricted rotational dynamics allows us to apply the eigendecompsition algorithm to delineate between longitudinal and transverse motions, ad detailed in Section I.2.

Last, to further verify our results of longitudinal and transverse motions, we calculate the MSDs for different axial directions in experiments. The MSDs along the major axis of the rods are much faster than those along the direction perpendicular to the major axis in experiments (Fig. S12). More importantly, the transverse displacements are found to be highly subdiffusive, indicating strong transverse localization. The residual slight increase of MSD with time (MSD $\sim t^{0.2}$ empirically) likely reflects limited motions such as the thermal fluctuation of the polymer mesh[42]; in contrast, the parallel displacements are approximate to linear in elapsed time (MSD $\sim t^{1}$ empirically). This is consistent with the results of some previous works[42,43] and corroborates the restricted transverse dynamics.

VI. Supplementary tables

Table S1. Some recent demonstrations of various bacteria in different mucus

Mucus	a_x	Bacterium	d	d/a_x	Reference
Colonic mucus	255nm	*Escherichia Coli*	250-500nm	1.0-1.9	44, 45
Intestinal mucus	240nm	*Vibrio cholerae*	500-800nm	2.1-3.3	46, 47
Porcine gastric mucin	525nm	rod-shaped *H. pylori*	500-1000nm	0.95-1.9	48
Gastric mucin	250nm	helical-shaped *H. pylori*	500nm	2.0	49, 50
Airway mucus	500nm	*Pseudomonas aeruginosa*	500-1000nm	1.0-2.0	51, 52
Airway mucus	500nm	*M. Tuberculosis*	500nm	1.0	53, 54
Airway mucus	500nm	*Porphyromonas*	500-800nm	1.0-1.6	55
Airway mucus	500nm	*Haemophilus influenzae*	300nm	0.60	56, 57
Airway mucus	500nm	*Escherichia Coli*	250-500nm	0.5-1.0	45, 58
Airway mucus	500nm	*Acinetobacter*	900nm	1.8	46, 49, 60
Cow cervical mucus	900nm	*Bacillus subtilis*	900nm	1.0	61, 62

Table S2. Storage modulus, loss modulus and mesh size for prepared hydrogels

Sample ID	Storage Modulus G' (kPa)	Loss Modulus G'' (kPa)	Modulus G (kPa)	Mesh size a_x (nm)
1	0.40	0.18	0.44	23.9
2	0.59	0.12	0.61	20.2
3	0.63	0.12	0.64	19.8
4	0.58	0.09	0.59	21.4
5	0.64	0.04	0.64	19.5
		Average	0.58 ± 0.08	21.0 ± 1.8

Table S3. Average and standard error of lengths and diameters of Au-NRs in experiments

Sample ID	01	02	03	04	05	06
Length(nm)	30.6 ± 3.4	42.7 ± 3.9	45.3 ± 4.9	52.5 ± 5.6	55.8 ± 6.7	61.4 ± 7.7
Diameter(nm)	18.2 ± 1.3	18.7 ± 0.9	19.7 ± 2.2	19.6 ± 1.7	19.9 ± 1.1	18.3 ± 1.9

Table S4. Mesh size distribution of typical biomacromolecular networks

Biomacromolecular Network	Mesh Size a_x	Standard deviation σ_{ax}	Coefficient of variation CV	Reference
Rat Intestinal Mucus	186nm	93.18nm	0.50	63
Collagen gel	7.0μm	1.0μm	0.14	64
Human Cervicovaginal Mucus	340 nm	70nm	0.21	65
Human Cervical Mucus	0.9 μm	0.7 μm	0.78	62

VII. Supplementary figures

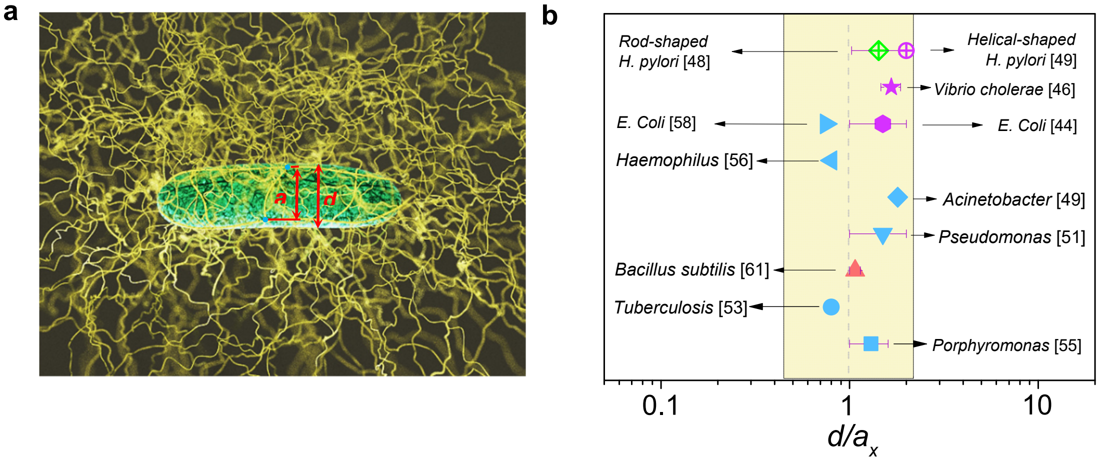

Fig. S1. (a) Schematic representation of a bacterium in the biomacromolecular network, where d and a_x denote respectively the diameter of the rodlike bacterium and the averaged mesh size of the network. (b) d/a_x for some recent demonstrations of various bacteria in different mucus. Each point with the same shape and color represent the same system in both panels. The point shape denotes bacterial type, as given in the panels. The color represents mucus type: (cyan) human airway mucus[49,51,53,55,56,58], (light red) cow cervical mucus[61], (purple) human gastrointestinal mucus[44,46,49], and (green) porcine gastric mucus[48].

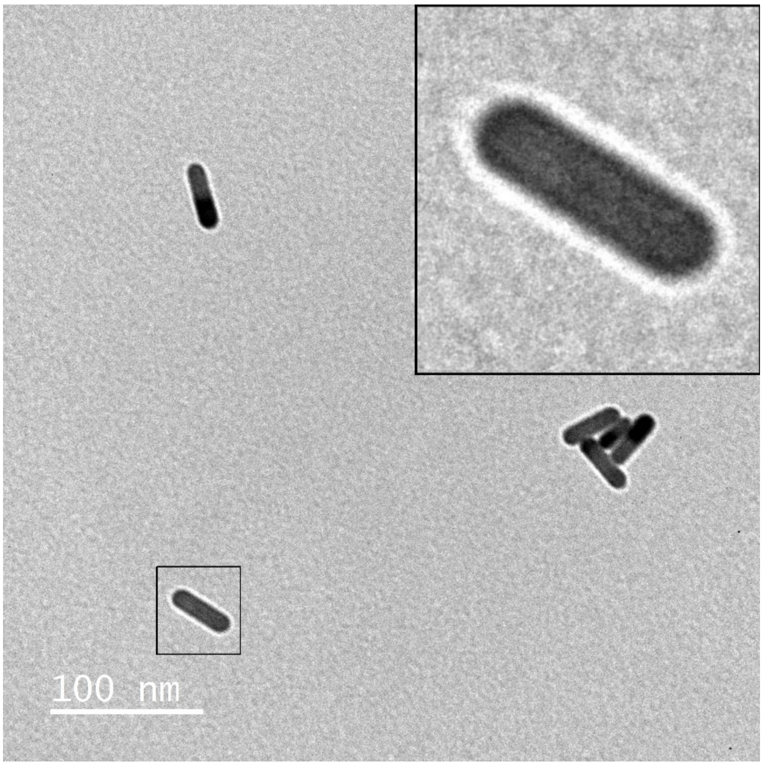

Fig. S2. A TEM image showing the representative Au-NRs used in experiments, where the length and diameter of the rod are 52.5 ± 5.6nm and 19.6 ± 1.7 nm, respectively. The inset shows a rod with enlarged size, highlighting the grafted layer, where the side length of the box is 50nm.

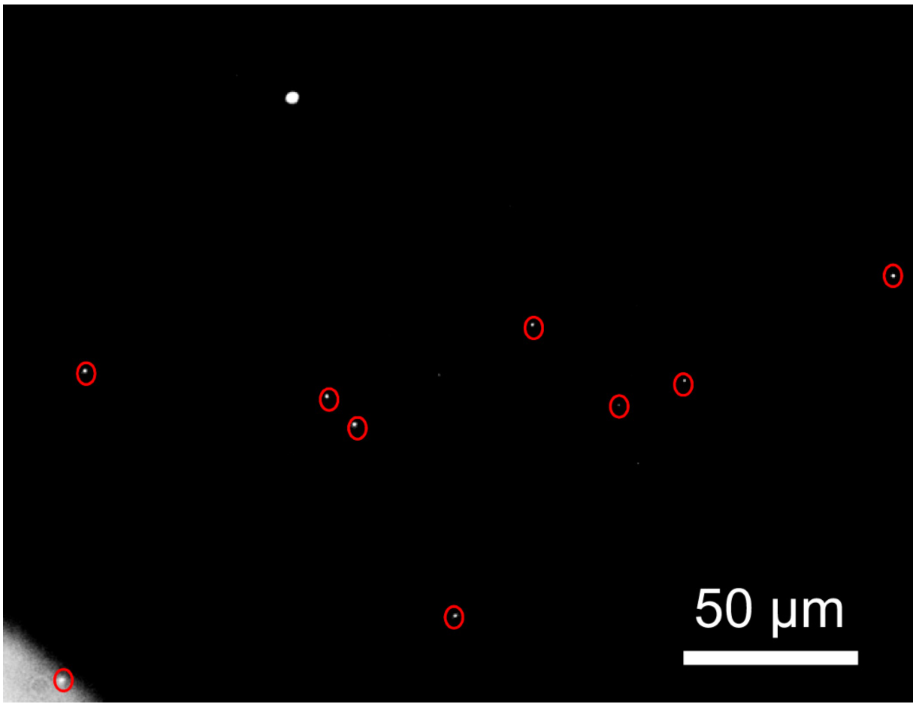

Fig. S3. A typical snapshot of rods in macromolecular networks in dark field microscopy, where the averaged length of rods is around 30.6 nm. The position of each rod is marked by a red circle.

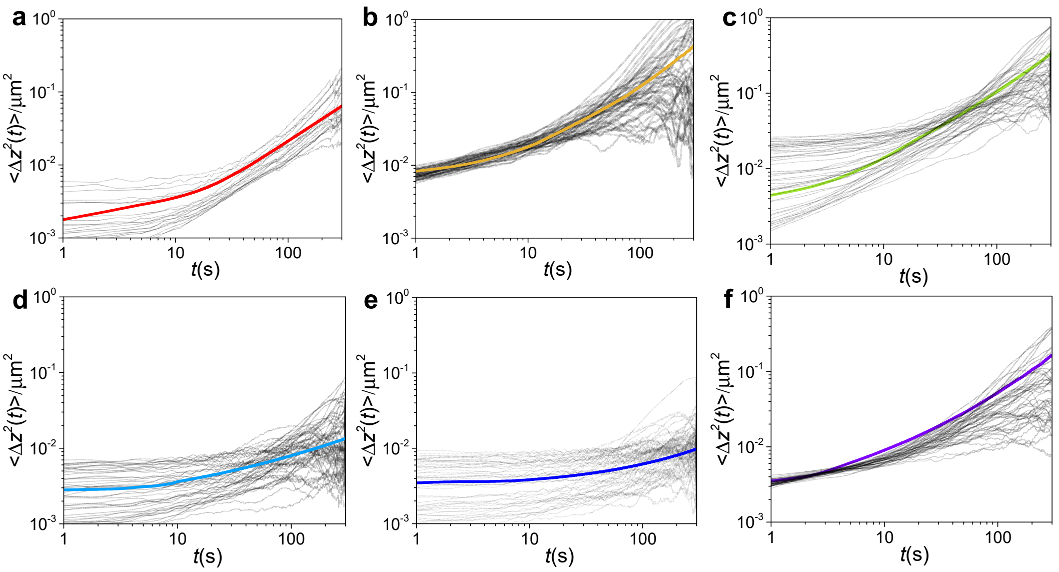

Fig. S4. Time-averaged (gray lines) and ensemble-averaged (colored lines) $<\Delta z^2(t)>$ plotted against time on log–log scales at different L/a_x: (a) $L/a_x = 1.46 \pm 0.16$, (b) $L/a_x = 2.02 \pm 0.18$, (c) $L/a_x = 2.16\pm0.24$, (d) $L/a_x = 2.51\pm0.27$, (e) $L/a_x = 2.63\pm0.31$ and (f) $L/a_x = 2.97\pm0.35$. Here $a_x \approx 21.0$ nm and $d/a_x \approx 1.0$.

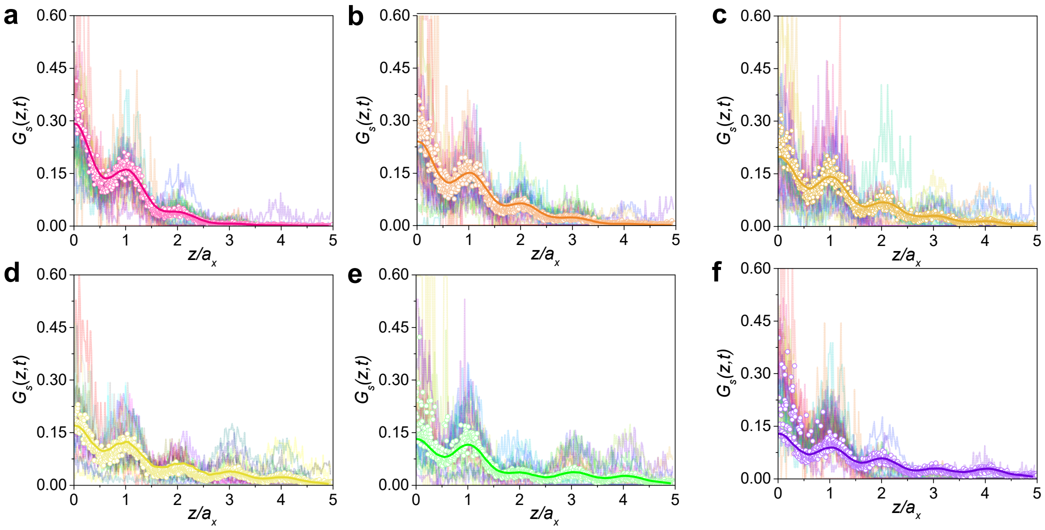

Fig. S5. The DPDFs obtained directly from the experimental measurements (half-transparent colored lines), the ensemble-averaged DPDFs (colored circles) and the smoothed lines for these circles (colored thick lines) of experimental $G_s(z, t)$ of rods with $L/a_x = 2.51 \pm 0.27$ at different times: (**a**) 220s (**b**) 380s (**c**) 500s (**d**) 650s (**e**) 750s and (**f**) 900s. The color of the half-transparent lines marks different particles.

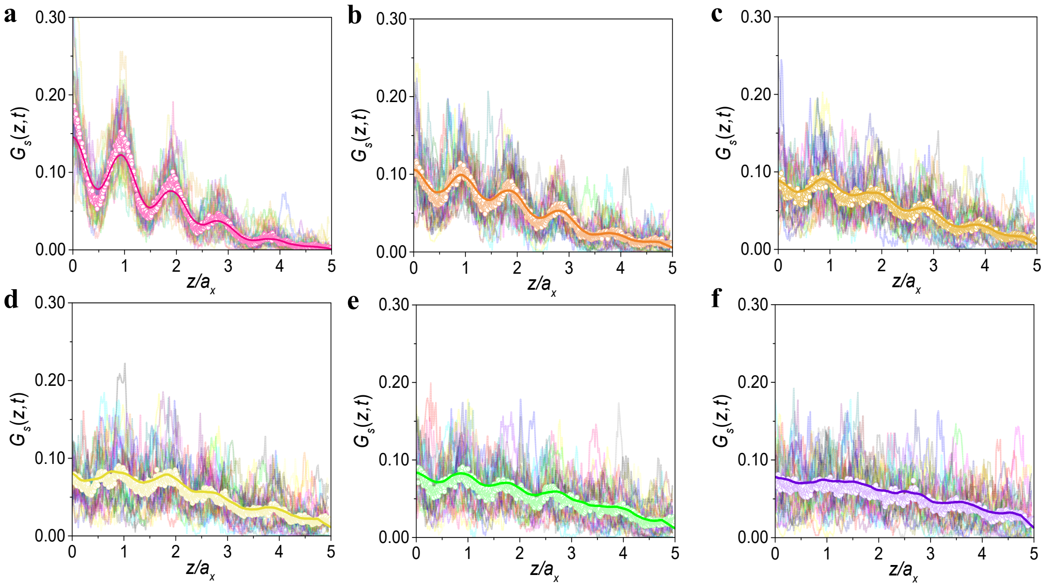

Fig. S6. The DPDFs obtained directly from the experimental measurements (half-transparent colored lines), the ensemble-averaged DPDFs (colored circles) and the smoothed lines for these circles (colored thick lines) of experimental $G_s(z, t)$ of rods with $L/a_x = 2.97 \pm 0.35$ at different times: (**a**) 220s (**b**) 380s (**c**) 500s (**d**) 650s (**e**) 750s and (**f**) 900s. The color of the half-transparent lines marks different particles.

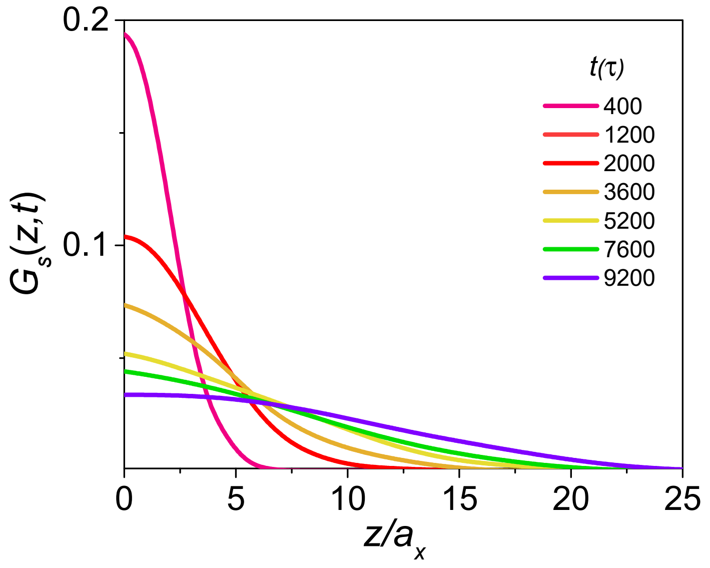

Fig. S7. The displacement probability distribution function $G_s(z, t)$ of Brownian motion.

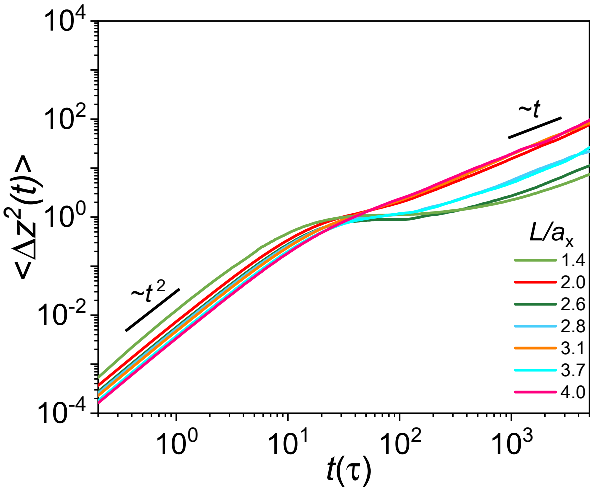

Fig. S8. $\langle \Delta z^2(t) \rangle$ for different L/a_x at d/a_x = 1.4 when a_{rp} = 30.

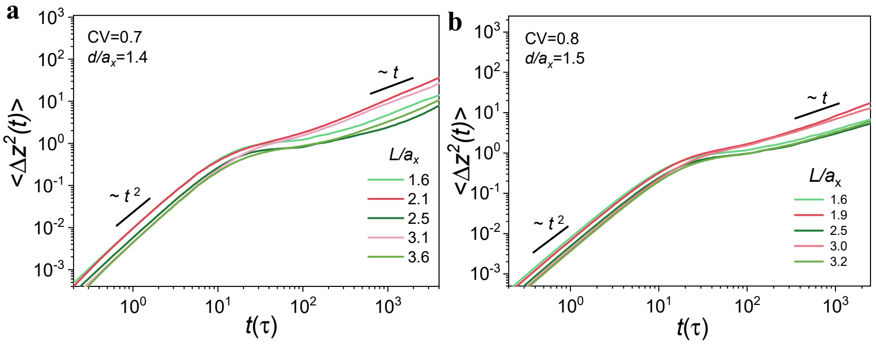

Fig. S9. $\langle \Delta z^2(t) \rangle$ for different L/a_x at (**a**) d/a_x = 1.4, CV=0.7, and (**b**) d/a_x = 1.5, CV=0.8.

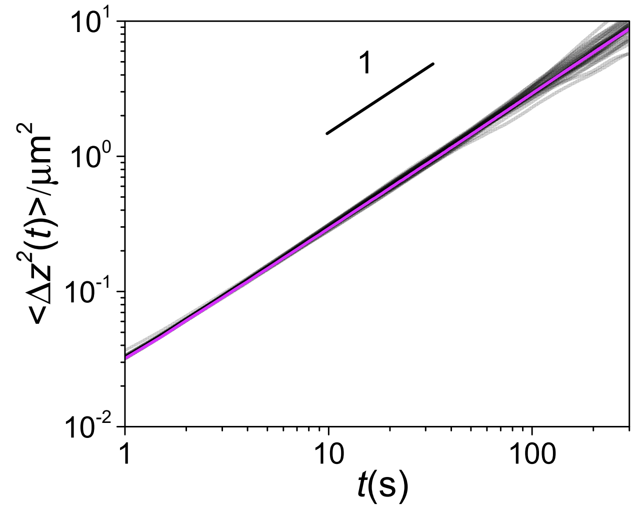

Fig. S10. Time-averaged $\langle \Delta z^2(t) \rangle$ (gray lines) and ensemble-averaged $\langle \Delta z^2(t) \rangle$ for 10800s (magenta line) plotted against time on the log–log scale, where d / a_x = 1.0 and L / a_x = 2.0.

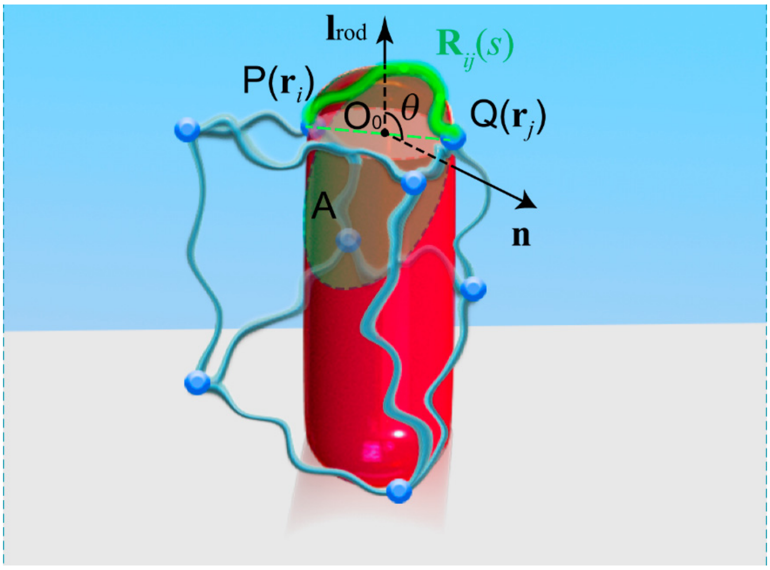

Fig. S11. Schematic of the deformation of network strands in contact with a rod, where the path of the strand $\mathbf{R}_{ij}(s)$ is colored by green curve and the translucent green plane represents the A plane which is an osculating plane (with the normal vector $\mathbf{n}$) passing through the cross-links P and Q with positional vectors $\mathbf{r}_i$ and $\mathbf{r}_j$.

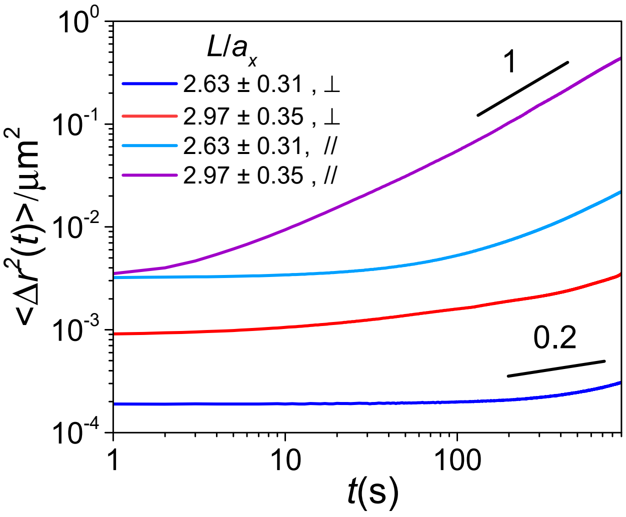

Fig. S12. The mean square displacement of the center of mass $\langle \Delta r^2(t) \rangle$ in different axial directions for rods with different lengths. (//) parallel to the major axis; (⊥) perpendicular to the major axis.

VIII. Supplementary References

1. Harris., J. M. & Chess, R. B. Effect of pegylation on pharmaceuticals. *Nat. Rev. Drug Discover.* **2**, 214-221(2023).
2. Hagel, V., Haraszti, T. & Boehm, H. Diffusion and interaction in PEG-DA hydrogels. *Biointerphases* **8**, 36 (2013).
3. Rose, K. A., Gogotsi, N., Galarraga, J. H., Burdick, J. A., Murray, C. B., Lee, D. & Composto, R. J. Shape anisotropy enhances nanoparticle dynamics in nearly homogeneous hydrogels. *Macromolecules* **55**, 8514-8523 (2022).
4. Canal, T. & Peppas, N. A. Correlation between mesh size and equilibrium degree of swelling of polymeric networks. *J. Biomed. Mater. Res.* **23**, 1183-1193 (1989).
5. Rehmann, M. S., Skeens, K. M., Kharkar, P. M., Ford, E. M., Maverakis, E., Lee, K. H. & Kloxin, A. M. Tuning and predicting mesh size and protein release from step growth hydrogels. *Biomacromolecules* **18**, 3131-3142 (2017).
6. Phelps, E. A., Enemchukwu, N. O., Fiore, V. F., Sy, J. C., Murthy, N., Sulchek, T. A., Barker, T. H. & García, A. J. Maleimide cross-linked bioactive PEG hydrogel exhibits improved reaction kinetics and cross-linking for cell encapsulation and in situ delivery. *Adv. Mater.* **24**, 64-70 (2011).
7. Lou, J. & Mooney, D. J. Chemical strategies to engineer hydrogels for cell culture. *Nat. Rev. Chem.* **6**, 726-744 (2022).
8. Schindelin, J., Arganda-Carreras, I., Frise, E., Kaynig, V., Longair, M., Pietzsch, T., Preibisch, S., Rueden, C., Saalfeld, S., Schmid, B., Tinevez, J.-Y., White, D. J.,

Hartenstein, V., Eliceiri, K., Tomancak, P. & Cardona, A. Fiji: An open-source platform for biological-image analysis. *Nat. Methods* **9**, 676-682 (2012).

9. Chazot, C. A. C., Nagelberg, S., Rowlands, C. J., Scherer, M. R. J., Coropceanu, I., Broderick, K., Kim, Y., Bawendi, M. G., So, P. T. C., Kolle, M. Luminescent surfaces with tailored angular emission for compact dark-field imaging devices. *Nat. Photon.* **14**, 310-315 (2020).
10. Park, Y., Shin, S., Jin, H., Park, J., Hong, Y., Choi, J., Jung, B., Song, H. & Seo, D. Single-molecule rotation for egfr conformational dynamics in live cells. *J Am. Chem. Soc.* **140**, 15161-15165 (2018).
11. Wong, I. Y., Gardel, M. L., Reichman, D. R., Weeks, E. R., Valentine, M. T., Bausch, A. R. & Weitz, D. A. Anomalous diffusion probes microstructure dynamics of entangled f-actin networks. *Phys. Rev. Lett.* **92**, 178101 (2004).
12. Allan, D. B., Caswell, T., Keim, N. C., van der Wel, C. M. & Verweij, R. W. *Soft-Matter/Trackpy: Trackpy v0.5.0*, Zenodo.
13. Crocker, J. C. & Grier, D. G. Methods of digital video microscopy for colloidal studies. *J. Colloid Interface Sci.* **179**, 298-310 (1996).
14. Schuster, B. S., Ensign, L. M., Allan, D. B., Suk, J. S. & Hanes, J. Particle tracking in drug and gene delivery research: state-of-the-art applications and methods. *Adv. Drug Deliver. Rev.* **91**, 70-91 (2015).
15. Lindén, M., Ćurić, V., Amselem, E. & Elf, J. Pointwise error estimates in localization microscopy. *Nat. Commun.* **8**, 11515 (2017).

16. Spillane, K. M., Ortega-Arroyo, J., De Wit, G., Eggeling, C., Ewers, H., Wallace, M. I. & Kukura, P. High-speed single-particle tracking of gm1 in model membranes reveals anomalous diffusion due to interleaflet coupling and molecular pinning. *Nano Lett.* **14**, 5390-5397 (2014).

17. Parsa, S., Guasto, J. S., Kishore, M., Ouellette, N. T., Gollub, J. P. & Voth, G. A. Rotation and alignment of rods in two-dimensional chaotic flow. *Phys. Fluids* **23**, 043302 (2011).

18. Pumir, A. & Wilkinson, M. Orientation statistics of small particles in turbulence. *New J. Phys.* **13**, 093030 (2011).

19. Groot, R. D. & Warren, P. B. Dissipative particle dynamics: Bridging the gap between atomistic and mesoscopic simulation. *J. Chem. Phys.* **107**, 4423-4435 (1997).

20. Yong, X., Kuksenok, O., Matyjaszewski, K. & Balazs, A. C. Harnessing interfacially-active nanorods to regenerate severed polymer gels. *Nano Lett.* **13**, 6269-6274 (2013).

21. Raos, G. & Casalegno, M. Nonequilibrium simulations of filled polymer networks: searching for the origins of reinforcement and nonlinearity. *J. Chem. Phys.* **134**, 054902 (2011).

22. Xu, Z., Dai, X., Bu, X., Yang, Y., Zhang, X., Man, X., Zhang, X., Doi, M. & Yan, L.-T. Enhanced heterogeneous diffusion of nanoparticles in semiflexible networks. *ACS Nano* **15**, 4608-4616 (2021).

23. Rubinstein, M. & Colby, R. *Polymer Physics* (Oxford University Press, 2003).

24. Nikunen, P., Vattulainen, I. & Karttunen, M. Reptational dynamics in dissipative particle dynamics simulations of polymer melts. *Phys. Rev. E* **75**, 036713 (2007).

25. Swope, W. C., Andersen, H. C., Berens, P. H. & Wilson, K. R. A computer simulation method for the calculation of equilibrium constants for the formation of physical clusters of molecules: Application to small water clusters. *J. Chem. Phys.* **76**, 637-649 (1982).

26. Persson, P.-O. & Strang, G. A. Simple mesh generator in MATLAB. *SIAM Rev.* **46**, 329-345 (2004).

27. Miller, T. F., Eleftheriou, M., Pattnaik, P., Ndirango, A., Newns, D. & Martyna, G. J. Symplectic quaternion scheme for biophysical molecular dynamics. *J. Chem. Phys.* **116,** 8649-8659 (2002).

28. Yang, K. & Ma, Y.-Q. Computer simulation of the translocation of nanoparticles with different shapes across a lipid bilayer. *Nat. Nanotechnol.* **5**, 579-583 (2010).

29. Calvaresi, M., Dallavalle, M. & Zerbetto, F. Wrapping nanotubes with micelles, hemimicelles, and cylindrical micelles. *Small* **5**, 2191-2198 (2009).

30. Deam, R. T & Edwards, S. F. The theory of rubber elasticity. *Phys. Trans. R. Soc.* **280**, 317-353 (1976).

31. Schmid, F. Self-consistent field approach for cross-linked copolymer materials. *Phys. Rev. Lett.* **111**, 028303 (2013).

32. Graham, R. An efficient algorithm for determining the convex hull of a finite planar set. *Info. Pro. Lett.* **1**, 132-133 (1972).

33. Montroll, E. W. & Weiss, G. H. Random Walks on Lattices. II. *J. Math. Phys.* **6**, 167-181 (1965).

34. Metzler, R. & Klafter, J. The random walk's guide to anomalous diffusion: a fractional dynamics approach. *Phys. Rep.* **339**, 1-77 (2000).

35. Cohen, A. E. Control of nanoparticles with arbitrary two-dimensional force fields. *Phys. Rev. Lett.* **94**, 118102 (2005).

36. Mel'nikov, V. The Kramersrs problem: fifty years of development. *Phys. Rep.* **209**, 1-71 (2009).

37. Dechant, A., Kindermann, F., Widera, A. & Lutz, E. Continuous-time random walk for a particle in a periodic potential. *Phys. Rev. Lett.* **123**, 070602 (2019).

38. Ślęzak, J. & Burov, S. From diffusion in compartmentalized media to non-Gaussian random walks. *Sci. Rep.* **11**, 5101 (2021).

39. Smith, M., Poling-Skutvik, R., Slim, A. H., Willson, R. C. & Conrad, J. C. Dynamics of flexible viruses in polymer solutions. *Macromolecules* **54**, 4557-4563 (2021).

40. Alam, S. & Mukhopadhyay, A. Translational and rotational diffusions of nanorods within semidilute and entangled polymer solutions. *Macromolecules* **47**, 6919-6924 (2014).

41. Poling-Skutvik, R., Krishnamoorti, R. & Conrad, J. C. Size-dependent dynamics of nanoparticles in unentangled polyelectrolyte solutions. *ACS Macro Lett.* **4**, 1169-1173 (2015).

42. Tsang, B., Dell, Z. E., Jiang, L., Schweizer, K. S. & Granick, S. Dynamic cross-correlations between entangled biofilaments as they diffuse. *Proc. Natl. Acad. Sci. U.S.A.* **114**, 3322 (2017).

43. Fakhri, N., Wessel, A. D., Willms, C., Pasquali, M., Klopfenstein, D. R., MacKintosh, F. C. & Schmidt, C. F. *Science* **344**, 1031 (2014).

44. Yildiz, H. M., Carlson, T. L., Goldstein, A. M. & Carrier, R. L. Mucus barriers to microparticles and microbes are altered in hirschsprung. *Macromol. Biosci.* **15**, 712-718 (2015).

45. M. Riley, *Size limits of very small microorganisms: proceedings of a workshop.* (National Academies Press Washington DC, 1999).

46. Nhu, N. T. Q., Lee, J. S., Wang, H. J. & Dufour Y. S. Alkaline pH increases swimming speed and facilitates mucus penetration for vibrio cholerae. *J. Bacteriol.* **203**, e00607 (2021).

47. Blue, D. E. & Schmitt, B. H. *Microbiology for the Surgical Pathologist. In Essentials of Anatomic Pathology* (Springer Science & Business Media, 2011).

48. Constantino, M. A., Jabbarzadeh, M., Fu, H. C. & Bansil, R. Helical and rod-shaped bacteria swim in helical trajectories with little additional propulsion from helical shape. *Sci. Adv.* **2**, e1601661 (2016).

49. Pelaseyed, T., Bergström, J. H., Gustafsson, J. K., Ermund, A., Birchenough, G. M. H., Schütte, A., Van der Post, S., Svensson, F., Rodríguez-Piñeiro, A. M., Nyström, E. E. L., Wising, C., Johansson, M. E. V. & Hansson, G. C. The mucus and mucins of the goblet cells and enterocytes provide the first defense line of the

gastrointestinal tract and interact with the immune system. *Immunol. Rev.* **260**, 8-20 (2014).

50. Bansil, R., Celli, J. P., Hardcastle, J. M. & Turner, B. S. The influence of mucus microstructure and rheology in helicobacter pylori infection. *Front. Immunol.* **4**, 310 (2013).

51. Duncan, G. A., Jung, J., Joseph, A., Thaxton, A. L., West, N. E., Boyle, M. P., Hanes, J. & Suk, J. S. Microstructural alterations of sputum in cystic fibrosis lung disease. *JCI Insight* **1**, e88198 (2016).

52. Diggle, S. P. & Whiteley, M. Microbe profile: Pseudomonas aeruginosa: opportunistic pathogen and lab rat. *Microbiology* **166**, 30-33 (2020).

53. Vijay, S., Hai, H. T., Thu, D. D. A., Johnson, E., Pielach, A., Phu, N. H., Thwaites, G. E. & Thuong, N. T. T. Ultrastructural analysis of cell envelope and accumulation of lipid inclusions in clinical mycobacterium tuberculosis isolates from sputum, oxidative stress, and iron deficiency. *Front. Microbio.* **8**, 2681 (2018).

54. Percival, S. L. & Williams, D. W. *Microbiology of Waterborne Diseases.* (Elsevier, 2014).

55. Durack, J., Lynch, S. V., Nariya, S., Bhakta, N. R., Beigelman, A., Castro, M., Dyer, A.-M., Israel, E., Kraft, M., Martin, R. J., Mauger, D. T., Rosenberg, S. R., Sharp-King, T., White, S. R., Woodruff, P. G., Avila, P. C., Denlinger, L. C., Holguin, F., Lazarus, S. C., Lugogo, N., Moore, W. C., Peters, S. P., Que, L., Smith, L. J., Sorkness, C. A., Wechsler, M. E., Wenzel, S. E., Boushey, H. A. & Huang,

Y. J. Features of the bronchial bacterial microbiome associated with atopy, asthma, and responsiveness to inhaled corticosteroid treatment. *J. Allergy Clin. Immunol.* **140**, 63-75 (2017).

56. Fahy, J. V. & Dickey, B. F. Airway mucus function and dysfunction. *N. Engl. J. Med.* **363**, 2233-2247 (2010).
57. Waksman, G., Caparon, M. & Hultgren, S. *Structural Biology of Bacterial Pathogenesis.* (ASM Press, 2005).
58. Matsui, H., Verghese, M. W., Kesimer, M., Schwab, U. E., Randell, S. H., Sheehan, J. K., Grubb, B. R. & Boucher, R. C. Reduced three-dimensional motility in dehydrated airway mucus prevents neutrophil capture and killing bacteria on airway epithelial surfaces. *J. Immunol.* **175**, 1090-1099 (2005).
59. Yamada, K., Morinaga, Y., Yanagihara, K., Kaku, N., Harada, Y., Uno, N., Nakamura, S., Imamura, Y., Hasegawa, H., Miyazaki, T., Izumikawa, K., Kakeya, H., Mikamo, H. & Kohno, S. Azithromycin inhibits muc5ac induction via multidrug-resistant acinetobacter baumannii in human airway epithelial cells. *Pulm. Pharmacol. Ther.* **28**, 165-170 (2014).
60. Blackburn, C. de W. *Food Spoilage Microorganisms.* (Woodhead Publishing, 2006).
61. Figueroa-Morales, N., Dominguez-Rubio, L., Ott, T. L. & Aranson, I. S. Mechanical shear controls bacterial penetration in mucus. *Sci. Rep.* **9**, 9713 (2019).

62. Leuschner, R. G., Ferdinando, D. P. & Lillford, P. J. Structural analysis of spores of bacillus subtilis during germination and outgrowth. *Colloids. Surf. B Biointerfaces* **19**, 31-41 (2000).

63. Yu, M., Wang, J., Yang, Y., Zhu, C., Su, Q., Guo, S., Sun, J., Gan, Y., Shi, X. & Gao, H. Rotation-facilitated rapid transport of nanorods in mucosal tissues. *Nano Lett.* **16**, 7176-7182 (2016).

64. Fischer, T., Hayn, A. & Mierke, C. T. Fast and reliable advanced two-step pore-size analysis of biomimetic 3d extracellular matrix scaffolds. *Sci. Rep.* **9**, 8352 (2019).

65. Lai, S. K., Wang, Y.-Y., Hida, K., Cone, R. & Hanes, J. Nanoparticles reveal that human cervicovaginal mucus is riddled with pores larger than viruses. *Proc. Natl. Acad. Sci. U.S.A.* **107**, 598-603 (2009).
